# The World’s First Acne Dysbiosis-like Model of Human 3D Ex Vivo Sebaceous Gland Colonized with *Cutibacterium acnes* and *Staphylococcus epidermidis*

**DOI:** 10.3390/microorganisms11092183

**Published:** 2023-08-29

**Authors:** Nico Forraz, Cécile Bize, Anne-Laure Desroches, Clément Milet, Pauline Payen, Pauline Chanut, Catherine Kern, Christine Garcia, Colin McGuckin

**Affiliations:** 1 CTISkin Department, CTIBIOTECH, 69330 Meyzieu, France; nico.forraz@ctibiotech.com (N.F.); annelaure.desroches@ctibiotech.com (A.-L.D.); clement.milet@ctibiotech.com (C.M.); pauline.payen@ctibiotech.com (P.P.); 2 Seppic Research & Innovation, 92250 La Garenne Colombes, France; cecile.bize@airliquide.com (C.B.); catherine.kern@airliquide.com (C.K.); christine.garcia@airliquide.com (C.G.)

**Keywords:** acne, sebocytes, sebaceous glands, bacteria, sebum, microbiota, skin, tissue engineering

## Abstract

Acne-prone skin is associated with dysbiosis involving *Cutibacterium acnes* (*C. acnes*) and *Staphylococcus epidermidis* (*S. epidermidis*) causing increased seborrhea in sebaceous glands (SG) and inflammation. Human primary sebocytes were cultivated using 1.10^6^ UFC/mL *C. acnes* Type IA (facial acne, ATCC6919) and/or 1.10^5^ UFC/mL *S. epidermidis* (unknown origin, ATCC12228) for 48 h in our SEB4GLN-optimized media without antibiotics. Bacteria and sebocytes were enumerated and assessed to determine their viability. Lipid production was imaged and quantified via Nile Red staining. SG with hair follicles were microdissected from healthy skin and cultured using 1.10^5^ UFC/mL *C. acnes* Type 1A and/or 1.10^4^ UFC/mL *S. epidermidis* (wild-type facial skin strain) through prior fixation and immunostaining for MC5R, *C. acnes* and nuclei (DAPI) via Z-stack confocal microscopy bioimaging (Leica SP5X & FIJI software, Version 2.9.0). *C. acnes* growth was not impacted when co-cultivated with sebocytes (2D) or SG (3D) models. Phylotype IA stimulated sebocyte lipid production, which had no impact on viability. The *S. epidermidis* reference strain overproliferated, inducing sebocyte mortality. For 3D SG model, culture conditions were optimized using a wild-type facial skin strain at a lower concentration, 1:10 ratio to *C. acnes,* reduced contact time, sequential inoculation and rinsing step. Bioimaging revealed strong *C. acnes* labeling in the active areas of the pilosebaceous unit. *S. epidermidis* formed biofilm, which was distributed across the SG via non-specific fluorescence imaging. We developed an innovative model of a sebaceous gland that mimics acne-prone skin with lipid overproduction and virulent phylotype IA *C. acnes* inoculation.

## 1. Introduction

Sebum is produced by the sebaceous glands, which are holocrine glands within the pilosebaceous unit composed of sebocytes. Undifferentiated sebocytes are located at the periphery of the gland proliferate, and some of them regularly start to differentiate and produce ever-more lipid droplets while migrating towards the center of the gland [1]. The final stage of sebocyte differentiation is cell death which allows the release of the cell contents into the hair follicle duct to which the gland is usually connected [2,3]. This constitutes sebum, which is mainly composed of neutral lipids, and the amount of sebum produced depends on both the proliferation of sebocytes and the stimulation of lipid metabolism [4].

Acne is a common chronic inflammatory disease estimated to affect 9% of people worldwide and 70% of teenagers, causing discomfort, as well as subsequent skin scarring and psychological distress and an emotional burden [5,6]. Although the triggers of acne are not fully understood, androgen-induced hypersecretion of sebum in the pilosebaceous unit is linked to recurrent inflammation, keratinization and bacterial colonization of *Cutibacterium acnes* (*C. acnes*), causing skin microbiota dysbiosis with commensal bacteria, such as *Staphylococcus epidermidis* (*S. epidermidis)* [6,7,8]. Due to the cosmetic strategies used in association with the microbial component, in this study, we described a new accurate 3D ex vivo human sebaceous gland model colonized with *C. acnes* and/or *S. epidermidis*. The objectives of the study were as follows: to validate co-culture conditions of human sebocytes grown in 2D monolayers in presence of *C. acnes* and/or *S. epidermidis*, followed by the development and validation of a robust reproducible 3D ex vivo human sebaceous gland model colonized with normal and dysbiotic microbiota.

## 2. Materials and Methods

### 2.1. Human Biological Specimen Collection

All skin samples were obtained from human donor via surgery following the provision of informed consent in accordance with applicable ethical guidelines and regulations in France. CTIBIOTECH has a double accreditation from the French Ministry to take charge of research used in the preparation (AC-2018-3243 and DC-2018-3242) and conservation of elements derived from the human body with a view to their transfer for scientific purposes. The operation was carried out according to the strictest hygiene rules. The sample was maintained in aseptic conditions throughout its journey.

### 2.2. Primary Human Sebocyts Isolation and 2D Monolayer Cell Culture

Primary human sebocytes were isolated from micro-dissected sebaceous glands via freshly resected ex vivo human skin biopsies, as previously described [9]. Epidermal and dermal tissue around the sebaceous gland were progressively removed using scalpels under a stereomicroscope in aseptic conditions. Sebaceous glands were then transferred using fibronectin-coated 6-well plates to create “air-liquid” ex vivo culture for sebocyte amplification in monolayers in CTIBIOTECH’s proprietary cell culture SEB4GLN.

### 2.3. Optimization of Experimental Conditions Required for Primary Human Sebocyte Co-Culturing with C. acnes and S. epidermidis

#### 2.3.1. Bacterial Strains Selection and Amplification

Two commercial reference strains were selected and amplified (Eurofins BactUp, St Priest, France): DSM 1897 (ATCC6919), which is a phylotype IA strain of *C. acnes* isolated from acne-prone facial skin, and CIP28.61 (ATCC12228), which is a strain of *S. epidermidis* of unknown origin (reference strain for FDA quality control tests). Strains were quality controlled for growth charts under optimal conditions, as shown below.

Suspensions of OD_600_ 0.5 to 1 were made and counted using a solid culture medium. Based on these data, the correlations between OD_600_ and colony forming units (CFU) were used to determine the parameters required to prepare the inoculum.

The following types of media were used to enumerate germs:

TSA Medium-Tryptone Soy Agar: a classic isolation medium used for the enumeration/isolation of undemanding germs;

COS Medium-Columbia Blood Agar: a classic isolation medium used for the enumeration/isolation of more demanding germs, as well as a classic medium used for anaerobic germs.

Both media were used in all pharmaceutical and cosmetic controls. Based on 48 h of pre-culturing on agar medium of the different micro-organisms, different inocula (IC) were tested to evaluate the survival rate of the micro-organisms in the sebocyte growth medium SEB4GLN per strain in duplicate.

After inoculation, counts were made at 2 h, 6 h, 24 h and 48 h of incubation at 37 °C +5% CO_2_. Enumerations were performed on the media and under the specific conditions for each strain, i.e., enumerations were performed on TSA agar for *S. epidermidis*, while enumerations were performed on COS agar for *C. acnes* (Table 1).

#### 2.3.2. Primary Human Sebocyte Co-Culturing with Bacterial Strains

Human primary sebocytes from facial skin were seeded at 30,000 cells per well in 6 well plates coated with fibronectin (Sigma-Aldrich, St Quentin Fallavier, France) and grown in SEB4GLN medium supplemented with linoleic acid (CTIBIOTECH, Meyzieu, France). The medium did not contain any antibiotics to avoid impeding the bacterial growth. After 5 days (now noted as Day 5, or D5), *C. acnes* and *S. epidermidis* were inoculated at concentrations of 2.46 × 10^5^ and 3.35 × 10^6^, respectively, in wells, with each well containing approximately 60,000 sebocytes based on the experimental conditions described below.

These values were based on those of previous studies using a ratio of 1:10 between *S. epidermidis* and *C. acnes* due to the predominant effect of *S. epidermidis* on *C. acnes* [10], as well as an optimized ratio of 1:100 between the SZ95 sebocyte cell line and *C. acnes* [11]. Test conditions were as follows: sebocytes alone, with added *C. acnes* alone, *S. epidermidis* alone, or both *C. acnes* and *S. epidermidis* conjointly added. The growth of *C. acnes* and *S. epidermidis* were also each evaluated alone, as well as conjointly. Testing of sebocytes alone was carried out to investigate the inherent growth of endogenous organisms as a control.

#### 2.3.3. Analysis of the Impact of Sebocytes on the Survival of Bacteria

At 48 h after the inoculation of the bacteria (now noted as Day 7 or D7), supernatants were collected, and the bacteria were counted according to the conditions described in Table 1. The counts of the culture supernatants were performed on both COS and TSA agars. Wells inoculated with only *S. epidermidis* only were aerobically plated on TSA, and wells containing only *C. acnes* were only anaerobically plated on COS. Wells simultaneously inoculated with both strains were plated on COS and TSA. On TSA agar, only the *S. epidermidis* strain could be counted. On COS agar, both strains were countable, but they were morphotypical differences between *C. acnes* and *S. epidermidis* strains.

The supernatants were diluted in saline solution (Peptone Salt Solution), and 100 µL of each dilution was spread in duplicate on 2 agars using a single-use rake. The concentration results for each strain indicated the concentration of the bacteria in the wells for each plate.

#### 2.3.4. Analysis of the Impact of Bacteria on the Survival of Sebocytes

At day 5 (D5, occurring just prior to the inoculation of the bacteria) and day 7 (D7, occurring at the end of culture), the culture supernatant was removed and replaced with culture medium containing 10% Alamar Blue (Fisher Scientific, Illkirch, France #DAL1025). After incubation for 1 h at 37 °C, the medium containing Alamar Blue was collected, and the cells were rinsed with Phosphate Buffered Saline solution (Fisher Scientific) before being returned to the culture medium and inoculated (D5) or fixed for further analysis (D7).

For each condition, the fluorescence intensity (FI) emitted by the solution containing Alamar Blue was measured using a fluorometer TECAN Spark^®^, Lyon, France) at a rate of 3 measurements per well and using the following wavelengths: excitation at 570 nm and emission at 585 nm.

#### 2.3.5. Analysis of the Impact of Bacteria on Lipid Production by Sebocytes

At the end of the experiment (D7), the sebocytes were fixed with 4% *w/v* formaldehyde (Sigma-Aldrich, France). Then, nuclei and cell lipids were labeled with 50 µg/mL of Hoechst solution and 7 µg/mL of Nile Red solution, respectively. The fluorescence intensity emitted by the cells was then measured in each well using a fluorometer (TECAN Spark^®^), with the following wavelengths being identified:

- Core (Hoechst): excitation = 356 nm and emission = 465 nm;
- Neutral lipids (Nile Red): excitation = 475 nm and emission = 530 nm;
- Total lipids (Nile Red): excitation = 520 nm and emission = 625 nm.

The raw values obtained were collected using the iControl software, Version 2.0.10 and exported to Excel for analysis.

Microscopic analysis was also performed using a fluorescence microscope (Eclipse Ti, Nikon, Tokyo, Japan) and processed using NIS-Elements BR 4.13.01 64-bit, Nikon, Tokyo, Japan. For each condition, photos were taken in a bright field and using different excitation and emission filters to reveal nuclei in blue (“DAPI”), neutral lipids in green (“FITC”) and total lipids in red (“TRITC”). A superposition of the 3 photos taken using the 3 types of filters revealed the lipid droplets in yellow.

### 2.4. Optimization of Experimental Conditions for 3D Ex Vivo Sebaceous Glands Using C. acnes and S. epidermidis

#### 2.4.1. Sebaceous Gland Extraction, Culture and Viability Assessment

In total, 24 sebaceous glands were isolated from a 46-year-old female under aseptic conditions, as previously described (CTISebogland, CTIBIOTECH, Meyzieu, France) [9].

**Figure 1:**
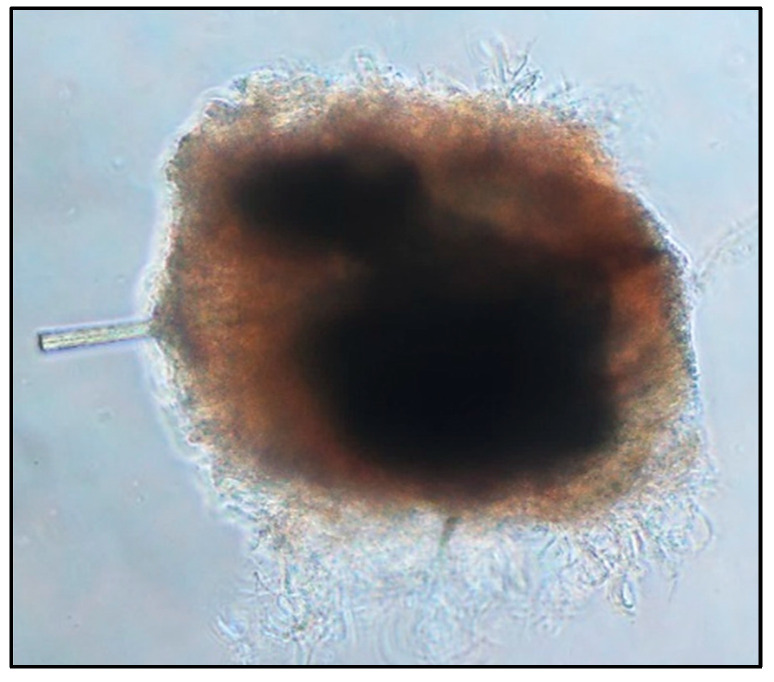
Example of an ex vivo sebaceous gland micro-dissected from human skin.

Sebaceous glands were obtained via microdissection with the assistance of a stereomicroscope (Leica, Nanterre, France) under aseptic conditions in a laminar flow hood from the skin sample (Figure 1). The sebaceous glands were then transferred to 48-well plates for ex vivo air–liquid survival culturing in 300 μL of antibiotic-free SEB4GLN media in a fibronectin-coated well (Fisher Scientific, Illkirch, France) at the “air-liquid” interface [9].

Processing of sebaceous glands was a laborious and delicate process that required much training in order to leave the delicate areas of the gland intact. Dense areas of the gland with large oil production capabilities were seen inside of the extracted tissue. In this case (though it is not always seen), a follicle passed through the gland.

Metabolic activity was assessed overnight on the day before inoculation of the bacteria into the model and 48 h afterward. The culture supernatant was removed and replaced with culture medium containing 10% Alamar Blue. After 12 h of incubation, for each duplicate condition, the fluorescence intensity (FI) emitted by the solution containing Alamar Blue was measured using a fluorometer (TECAN Spark^®^).

#### 2.4.2. Inoculation of Bacteria on the 3D Sebaceous Gland Model

**Figure 2:**
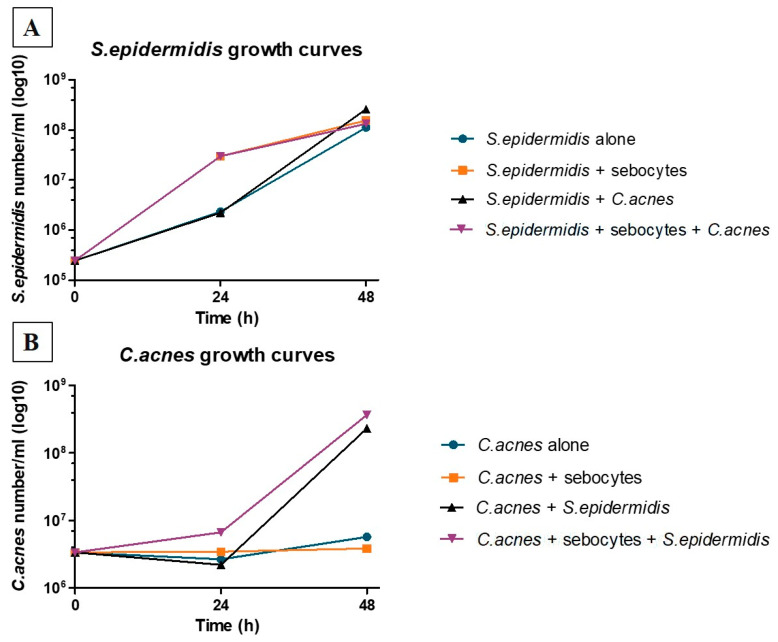
Evolution of the counts *S. epidermidis* (**A**) and C. *acnes* (**B**) in the presence or absence of sebocytes. The evaluation of strain growth showed differences between 24 and 48 h, with *C. acnes* requiring more time to separate based on the conditions presented. In contrast, in all cases, *S. epidermidis* achieved similar growth by 48 h.

Bacterial strains were selected, validated and amplified (Eurofins BactUp) using DSM 1897 (ATCC6919), which is a phylotype IA strain of *C. acnes* isolated from acne-prone facial skin and *S. epidermidis* derived from human facial sebaceous gland. The change from the CIP28.61 (ATCC12228) strain of *S. epidermidis* of unknown origin to a wild type of facial *S. epidermidis* was justified based on the overproliferation characteristics observed in the 2D monolayer sebocyte model (cf. Section 3.1, Figure 2).

Next, 3 days after extraction of the sebaceous glands, *S. epidermidis* and *C. acnes* were inoculated at concentrations of 1 × 10^4^ and 1 × 10^5^, respectively, in wells containing a single sebaceous gland. These values were established based on results derived from 2D monolayer co-culture and the literature showing the predominance of *S. epidermidis* over *C. acnes* in our experimental conditions.

**Scheme 1:**
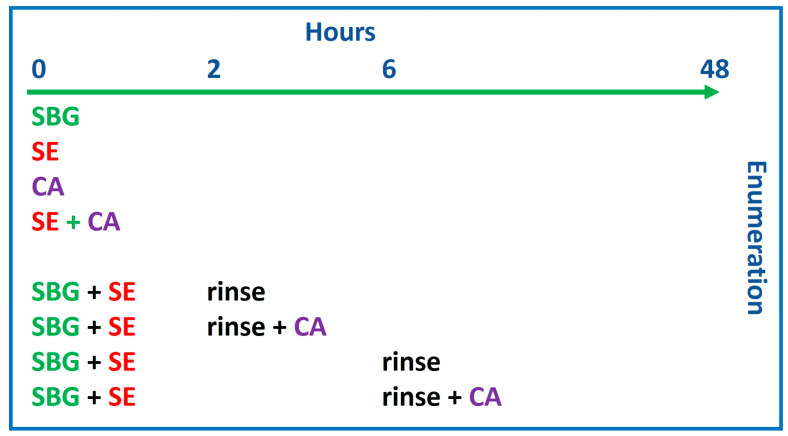
**A** representation of the timing of the treatments carried out in the study. SBG, sebaceous glands; SE, *S. epidermidis* 1 × 10^4^ CFU/mL; CA, *C. acnes* 1 × 10^5^ CFU/mL. SBG were tested 3 days after extraction for equilibration, which equated to Hour 0 of testing.

Then, 8 different experimental conditions were tested, as shown in Scheme 1.

After incubation for 48 h at 37 °C and 5% CO_2_, the supernatants were recovered for enumeration. Depending on the conditions, the counts of the culture supernatants were performed on one agar (COS or TSA) or simultaneously on both types of agars, as described in Section 2.3.1.

#### 2.4.3. Bio-Imaging of Microbiota on Sebaceous Glands

##### Immunostaining

Following Alamar Blue viability assay, the sebaceous glands were gently rinsed three times with PBS, fixed with 4% *w/v* formaldehyde (Sigma-Aldrich, St Quentin Fallavier, France) for 1 h and maintained in PBS. After rinsing, the samples underwent a permeabilization step necessary to allow the penetration of the antibodies.

Indirect immunostaining was then carried out to highlight the morphology of the sebaceous gland (MC5R, Abcam, Cambridge, UK) and its colonization by the microbiota (*C. acnes*, MBL International, Woburn, MA, USA).

Sebaceous glands were incubated 66 h prior to rinsing and incubation using secondary antibodies (Alexa Fluor 488 goat antirabbit IgG or Alexa Fluor 546—goat anti mouse IgG1, Fisher Scientific). Cells nuclei were counterstained with DAPI (4′,6-diamidino-2-phenylindole), which is a fluorescent molecule capable of binding strongly to DNA (Sigma-Aldrich).

##### Confocal Microscopy Bioimaging

Prior to confocal acquisition, the samples were placed in a 35-mm glass-bottomed petri dish covered with a coverslip. Image acquisition was performed via a Leica SP 5 X confocal microscope (Leica, Germany) with oil immersion objectives (0.5-mm magnification). Cells’ nuclei were labeled in blue (UV), MC5R were labeled in green (AlexaFluor 488, Thermofisher, Lyon, France) and, finally, *C. acnes* were labeled in red (AlexaFluor 546). Acquisition parameters (gain, laser power, etc.) were defined during the analysis and then uniformly applied to all of the conditions of the same magnification to allow image comparison. *S. epidermidis* showed up in the red channel as an autofluorescent signal. The optical focusing allows acquisition on a very precise focal plane to make a series of images at different depths in the sample called “Z-Stack” (every 2 to 3 µm). Three-dimensional reconstruction of Z-stacks, data processing and analysis were performed using ImageJ software and the FIJI module version 2.9.0 (NIH, Bethesda, Rockville, MD, USA).

### 2.5. Statistics

Differences in specific results were calculated via computer based on original data using One-Way ANOVA, Dunnett’s Multiple Comparison Test, Ordinary One-Way ANOVA and Tukey’s Multiple Comparison Test.

## 3. Results

### 3.1. S. epidermidis Inoculum Must Be Adjusted to Prevent Overproliferation in the Presence of C. acnes and Sebocytes

Following culturing with and without sebocytes, the TSA agar permitted the sole enumeration of *S. epidermidis*, as *C. acnes* does not multiply under those conditions. On COS agar, both strains could be counted and were morphotypically different, which allowed the differentiation between *C. acnes* and the sole enumeration of *S. epidermidis* strains.

*C. acnes* was maintained for 48 h without overgrowth, whether it was grown alone or in the presence of sebocytes, confirming that sebocyte culture conditions were suitable (without antibiotics) for the assay. Similar results were obtained in the presence of *S. epidermidis* during the first 24 h of culture after inoculation. Stimulation of *C. acnes* proliferation was generated by *S. epidermidis* between 24 and 48 h following inoculation. Significant growth of *S. epidermidis* occurred during the 48 h of culturing, and this outcome was observed in the presence or absence of *C. acnes* and/or sebocytes, indicating the absence of a significant effect of *C. acnes* and sebocytes on the growth of *S. epidermidis.* Since the selected *S. epidermidis* strain did not appear to be appropriate for the establishment of the desired model, an endogenous strain isolated from a human sebocyte gland was selected to perform the 3D sebaceous gland ex vivo assay (Figure 2).

### 3.2. Human Sebocytes Viability and Metabolism Are Negatively Affected by the Overproliferation of S. epidermidis, but Not by C. acnes

**Figure 3:**
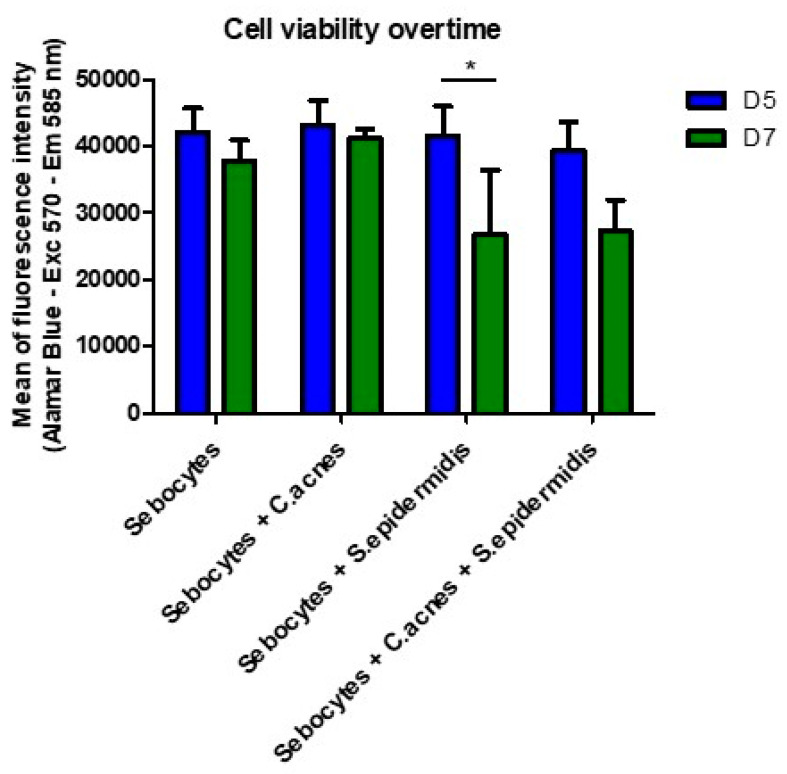
Comparison between cell viability under different culture or co-culture conditions of bacteria and sebocytes just before inoculation with bacteria (D5—day 5) or 48 h after inoculation (D7—day 7). *C. acnes* did not illicit particular changes compared to the sebocytes along the control, but the effect of *S. epidermidis* was more marked with or without *C. acnes*. * *p* value = 0.0309 One-Way ANOVA test (95% confidence interval—alpha + 0.05%) was used to perform statistical comparison between Day 5 and Day 7, with a reduction in viability occurring due to *S. epidermidis*. No statistical differences were recorded between all Day 5 values, as expected (same conditions), and the addition of *C. acnes* to *S. epidermidis* increased the viability in the statistically similar category.

**Figure 4:**
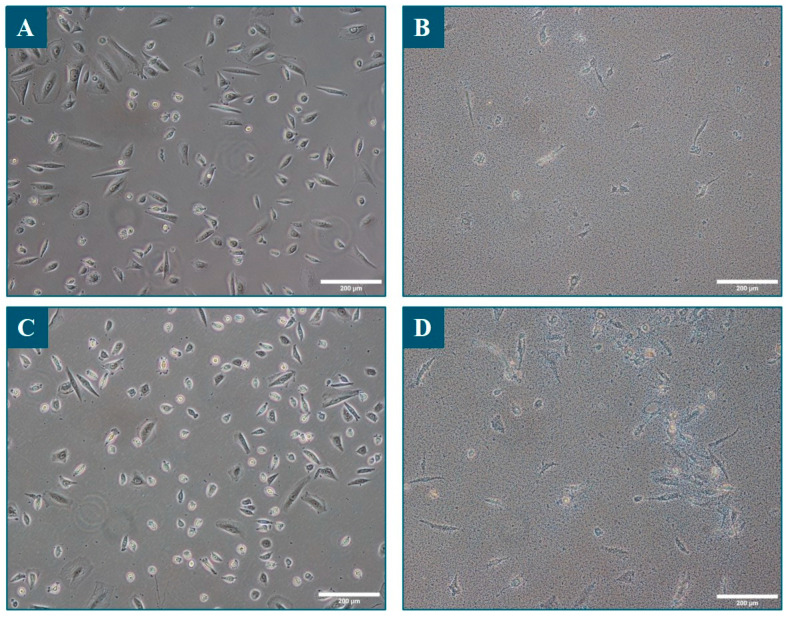
Bright-field microscopy of sebocytes grown with or without bacteria. Images were taken 48 h after the inoculation of the bacteria, just after removing the culture supernatant containing the bacteria and just before performing the Alamar Blue test. Panel (**A**): sebocytes only; Panel (**B**): sebocytes and *S. epidermidis*; Panel (**C**): sebocytes and *C. acnes*; Panel (**D**): sebocytes and *C. acnes* and *S. epidermidis.* Scale bar: 200 µm.

Sebocyte metabolism and viability were comparatively assessed prior to bacteria inoculation and 48 h post-inoculation via the Alamar Blue test. A good viability homogeneity in sebocyte numbers between all replicates and cell culture wells was observed at Day 5 (Figure 3), which was estimated to be 60,000 sebocytes per well. Next, 48 h after the inoculation of the bacteria, no major variation in this viability was observed in the control condition (sebocytes without bacterial inoculation) or after the inoculation of *C. acnes* alone. Furthermore, no signal was detected in the condition in which *C. acnes* was inoculated in the absence of sebocytes. Almost no bacteria were visible at the bottom of the culture plate (Figure 4C). It was, therefore, concluded that *C. acnes* did not have a particular positive or negative effect on sebocyte proliferation or survival. The particular strain was, therefore, selected for subsequent evolution of the model on 3D sebaceous glands.

Sebocyte metabolic activity and viability was decreased in the presence of *S. epidermidis*, regardless of whether *C. acnes* was present (Figure 3). Microscopic observations also showed that the sebocytes were much less numerous under these conditions and tended to detach, while many bacteria adhered to the bottom of the plate and onto the sebocytes (Figure 4B,D). The inoculated *S. epidermidis* strain, therefore, significantly decreased the viability of sebocytes and confirmed the need to select a skin-specific strain for progression to the 3D sebaceous gland model.

### 3.3. C. acnes Induced Total and Neutral Lipid Production by Human Sebocytes

**Figure 5:**
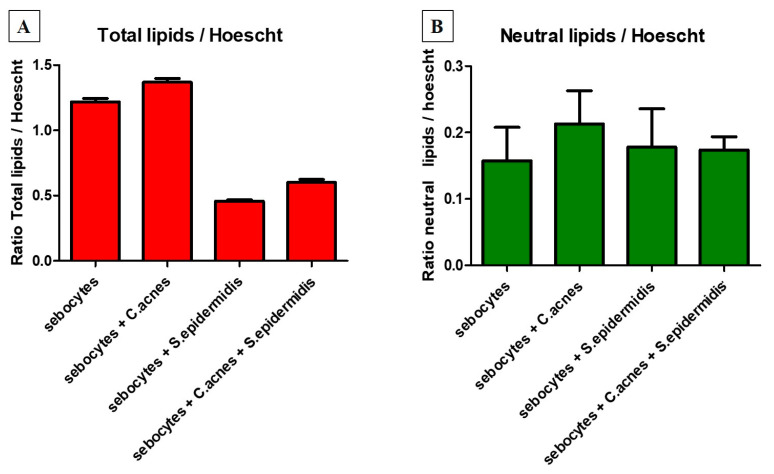
Comparison between fluorescence intensity ratios of Nile Red and Hoechst signals obtained using excitation and emission wavelengths of 520 and 625 nm for total lipids Panel (**A**) and 475 and 530 nm for neutral lipids Panel (**B**), as well as 356 and 465 nm for nuclei. CA: *C. acnes*; SE: *S. epidermidis.* Statistical analysis via One-way ANOVA and Dunnett’s Multiple Comparison Test (95% confidence interval; alpha = 0.05%) for Total Lipids showed *p* values of 0.0045 for sebocytes compared to sebocytes with added *C. acnes*, as well as 0.001 for sebocytes compared to adding *S. epidermidis* and *S. epidermidis* combined with *C. acnes*. No statistical differences were found for the Neutral Lipids.

Nile Red labels lipids and, depending on the excitation and emission wavelengths used, it reveals more neutral lipids (analyzed in Figure 5B; mainly constituting lipid droplets containing sebum), polar lipids (mainly constituting membranes) or total lipids (all lipids, analyzed in Figure 5A). Furthermore, Hoechst labels nuclei. The ratio of the fluorescence intensity of Nile Red to that of Hoechst, therefore, allows the quantity of lipids per cell to be estimated.

**Figure 6:**
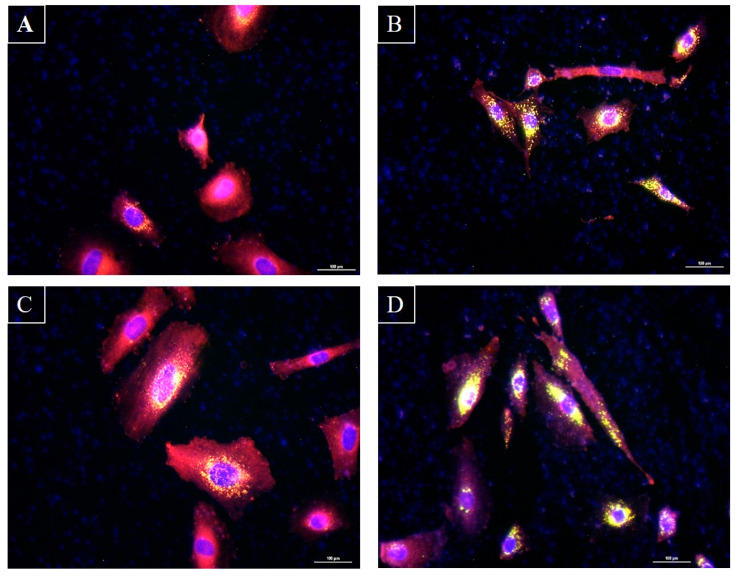
Microscopic observation of sebocytes cultured after fixation and labeling with Nile Red and Hoechst. Panel (**A**): Sebocytes alone. Panel (**B**): Sebocytes with *S. epidermidis*. Panel (**C**): Sebocytes with *C. acnes*. Panel (**D**): Sebocytes with *S. epidermidis* and *C. acnes*. Pictures were taken via an epifluorescence microscope. Hoechst marks the nuclei in blue, while Nile Red reveals neutral lipids in green and total lipids in red. Superimposing the two colors highlights the lipid droplets in yellow. Scale bar: 100 µm.

*C. acnes* inoculation alone with human sebocytes both quantitatively and qualitatively induced an increase in total and neutral lipids (constitutive of sebum; Figure 5A,B and Figure 6C) in the absence of *S. epidermidis*. *S. epidermidis* appeared to decrease total lipid production, which can be correlated with the deleterious effect it had on sebocyte viability. Interestingly, when combined with *C. acnes*, *S. epidermidis* maintained the decrease in neutral lipid secretion and slightly increased total lipid production when compared to the condition inoculated with *S. epidermidis* alone. Statistical evaluation appeared to confirm that *S. epidermidis* has a detrimental effect on total lipid production. These results were confirmed via fluorescence bioimaging (Figure 5B and Figure 6D).

### 3.4. C. acnes and S. epidermidis Strains and Inoculation Ratio Can Be Optimized for 3D Sebaceous Gland Ex Vivo Culture

Following observations made for 2D sebocyte cultures, a facial skin-specific strain of *S. epidermidis* (isolated from sebaceous gland) was isolated and amplified for the project to replace the CIP28.61 (ATCC12228) strain of *S. epidermidis* of unknown origin with high proliferation characteristics.

The bacterial counts summarized in Table 2 highlight that when *C. acnes* and *S. epidermidis* (facial skin strain) had the 10:1 ratio, the inoculum was able to observe bacterial proliferation in the 3D ex vivo sebaceous gland model. The counts further confirmed that the specific sebaceous gland culture conditions (fibronectin coating, SEB4GLN, 37 °C, 5% CO_2_) are classically favorable to a sebaceous gland/bacteria (*C. acnes*/*S. epidermidis*) co-culturing with a balanced ratio of inoculum “*S. epidermidis* 1× 10^4^ + *C. acnes* 1× 10^5^ ”. However, the final concentrations measured were found to be lower than the concentrations obtained from the method of individually inoculating the two strains (*S. epidermidis* 1× 10^4^ or *C. acnes* 1× 10^5^).

The concentrations obtained after rinsing the wells at 2 h and 6 h (*S. epidermidis* and rinsing at 2 h and *S. epidermidis* and rinsing at 6 h) confirmed the ability of this method to limit the potential overproliferation of S. *epidermidis*. The final concentration measured after 2 h (1.46 × 10^8^) is lower than the concentration noted after 6 h of contact. The concentration with rinsing at 6 h (4.57 × 10^8^) is also lower than the concentration measured after 48 h (7.10 × 10^8^).

The inoculation of *C. acnes* after rinsing also shows a certain proportionality between the final concentration measured and the contact time, which validates the following rinsing method:

- *C. acnes*: 1.09 × 10^9^;
- *S. epidermidis*: rinse at 2 h and C. acnes 1 × 10^5^ and after rinse: 2.71 × 10^8^;
- *S. epidermidis*: rinse at 6 h and C. acnes 1 × 10^5^ and after rinse: 1.79 × 10^8^.

**Figure 7:**
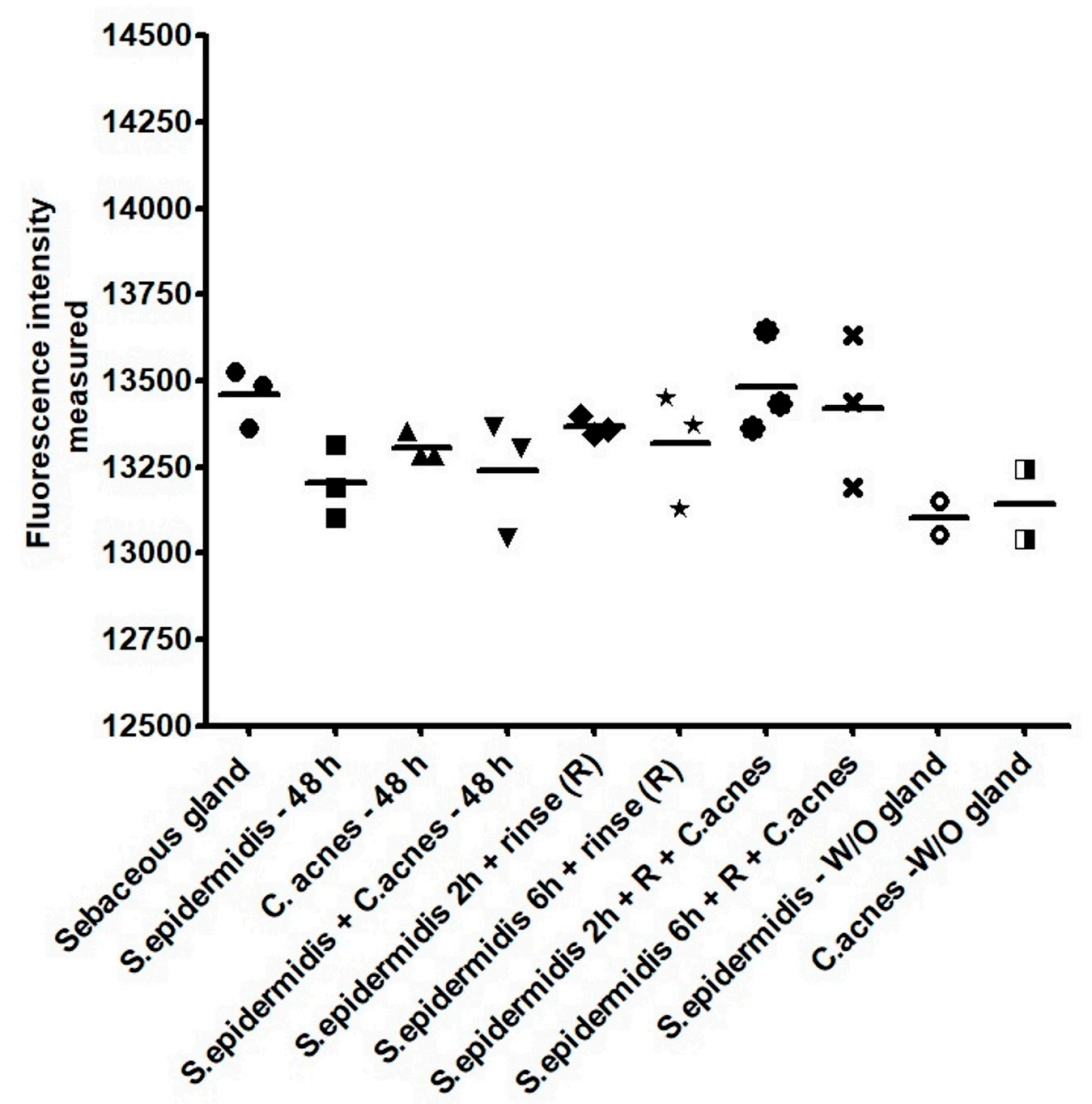
Alamar blue fluorescence intensity measured 48 h after the inoculation of the bacteria confirmed the preserved viability/metabolism of the sebaceous glands. The maintenance of viability during the time period chosen for culture was evaluated to ensure that cellular components of the sebaceous glands could continue activity and allow the evaluation of colonization. No major statistical differences were shown between these conditions.

Interestingly, this ratio of inoculation, regardless of the rinsing method, did not have any significant negative effect on the sebaceous gland’s overall metabolism or the viability, as highlighted by the Alamar blue fluorescence intensity with a rather homogeneous signal measured via spectrophotometry. Very strong increases in fluorescence intensity in all tested conditions were noted compared to the values detected before inoculation. All of the values obtained can be associated with the presence of the bacteria, rather than with the presence of the gland alone (Figure 7).

### 3.5. C. acnes Bacterial Load Can Be Imaged via 3D Confocal Microscopy of Sebaceous Glands

**Figure 8:**
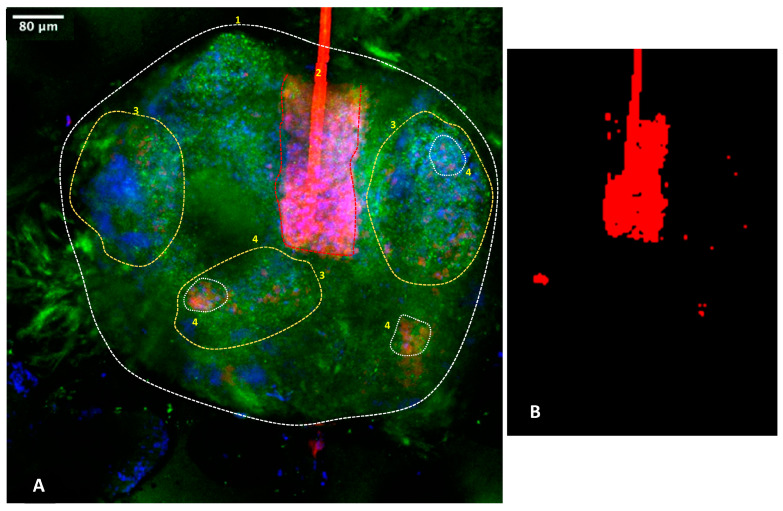
Representative 3D bioimaging of *C. acnes* on ex vivo sebaceous gland via confocal microscopy. Orientation has been corrected to the surface direction of the skin. Blue represents cell nuclei, green represents MC5R-positive sebocytes and red represents for *C. acnes* bacteria. Area 1: overall location of the gland. Area 2: hair follicle and hair sheath passing through gland with red autofluorescence of the follicle material and other *C. acnes red labelling in the lower bulb area*. Area 3: active areas of the gland. Area 4: examples of location of *C. acnes*. Panel (**A**): full gland. Panel (**B**): *C. acnes* areas labelled red. Localization of the *C. acnes* bacteria to active areas of the glands, including the hair follicles and gland lobes, is noted at areas 3 and 4. Supplementary Videos S1 and S2 available from the authors show further examples.

The analysis of confocal imaging was conducted to focus on the *C. acnes*, given the implications for future research. Figure 8 gives a summary diagram and Supplementary Materials available from the authors, including substantial additional data too large to be included one journal paper, indicating that *C. acnes* is a bacteria targeting specific active areas of the sebaceous gland, whereas the *S. epidermidis* can be located throughout the gland. The negative control without inocula did not show any *C. acnes* labeling, but the morphology of the gland (MC5R labelling, available from authors), as well as the nuclei, could be clearly identified (in blue). The positive control, which had inoculation of *C. acnes*, showed positive red staining localized at the center of the sebaceous gland active areas, particularly where final sebum production occurred. *C. acnes* was also detected with the same pattern in the active areas of both sebaceous glands and the hair follicles when hair follicles transversed the gland. *S. epidermidis* was imaged via non-specific fluorescence and located across the entire gland. The condition “*S. epidermidis 2 h +* rinse + *C. acnes*” provided the most interesting bioimaging of *C. acnes* labeling in the active part of the sebaceous gland (Figure 8 and Supplementary Videos S1 and S2). Rinsing after 2 h or 6 h was effective since we observed a decrease in non-specific labeling from the *S. epidermidis*.

## 4. Discussion

The involvement of microbiota on skin homeostasis is gaining much interest and provides a wealth of opportunity for innovation in dermocosmetics [12,13,14,15,16]. We previously reported 2D sebocyte monolayers in vitro methods combined with 3D ex vivo culture systems to perform sebaceous gland tissue engineering as a robust approach for the screening and development of innovative active ingredients, formulated products and medical/aesthetic devices for skincare [9]. This study investigated the optimization of culturing conditions to integrate two specific bacterial strains involved in acne dysbiosis, i.e., *S. epidermidis* and *C. acnes*, as well as, in particular, phylotype IA, which is known to be particularly virulent [17,18,19,20,21].

The challenge for any predictive in vitro/ex vivo human tissue bioassay is to be sufficiently predictive in order to mimic processes that happen in vivo in humans while providing robust measurable biological signals to understand the effects of products on skin systems [4,18,22,23].

In another sudy, the 2D model using primary sebocytes supplemented with acne dysbiosis bacterial strain *S. epidermidis* and *C. acnes* was successfully used to demonstrate the efficacy of *L. plantarum* in modulating and improving the host–skin microbiota interactions in subjects with mild-to-moderate papulopustular acne. The 2D model underlined the potential mechanisms of action of *L. plantarum* as an acne-mitigating agent targeting the underlying causes of inflammation and inhibiting the growth of acne-causing bacteria [24].

This study adopted a two-step approach with a progression from 2D primary human sebocyte co-culturing with bacteria to a 3D ex vivo sebaceous gland system for further investigation of microbiota on seborrhea.

Both the 2D and 3D approaches showed that human sebocytes and sebaceous glands could be successfully cultured with *C. acnes* and/or *S. epidermidis*. The origin of the strain choice is also important, particularly for *S. epidermidis*. Although we had initially hypothesized that the FDA reference CIP28.61 (ATCC 12228) of *S. epidermidis* (unknown origin) would be a good candidate for the model, its growth profile was rather intense, and adopting a facial skin-derived *S. epidermidis* strain was better suited to the 3D ex vivo sebaceous gland model. The sebocyte-friendly culture conditions enabled the inoculation of both bacterium types in 2D and 3D, as well as enabling lipid production measurement, viability assessment and bioimaging. Total and neutral (sebum) lipid neo synthesis induction by *C. acnes* could be measured efficiently with and without the presence of *S. epidermidis.* For a long time, *C. acnes* (particularly phylotype IA) was implicated in inflammatory acne, with elevated seborrhea, hyperkeratinization and induced hypoxia with the pilosebaceous gland unit and its epidermal pathophysiology previously being reported in reconstructed human epidermis models [25,26]. However, no difference in *C. acnes* load can be detected from healthy and acne-affected skin [7]. The dysbiosis observed acne skin microbiota can lead to the selection of specific *C. acnes* lineages, (phylotypes IA1/IA2) and a recent study confirmed that this could lead to production of Christie–Atkins–Munch–Petersen factor (CAMP)1 by *C. acnes* lineages with strong toll-like receptor (TLR)-2 binding activity to induce inflammation [26,27,28].

The doses and relative ratio of the inoculum between *C. acnes* and *S. epidermidis* (10:1) were paramount to maintaining the cultures over 48 h to reduce the risk of *S. epidermidis* taking over the artificial microbiota of the gland. From the results obtained and our observations, *C. acnes* is detected in the 3D ex vivo sebaceous gland model during a 48-h culture (*C. acnes* alone and *S. epidermidis 2 h and* rinse and *C. acnes).* The 3D model confirmed that during co-inoculation, *S. epidermidis* should be inoculated first and rinsed within 2 h prior to inoculation with *C. acnes*. Based on the growth characteristics observed, this observation was confirmed via the bioimaging of *S. epidermidis*, which induced an unspecific autofluorescence signal, probably due to the known ability of this bacteria to rapidly form biofilm [6,11,29]. Previous studies observed that *C. acnes* could stimulate biofilm formation by S. epidermidis [30,31,32,33], which could also be observed in the 3D ex vivo sebaceous gland model described in this study. Taken together, the results presented here confirmed the importance of using microbiota strain in co-culture with sebocytes to create a relevant model to investigate the efficacy of dermocosmetics and pharmaceutical products in acne-like models.

## 5. Conclusions

We developed innovative models of 2D in vitro primary sebocytes monolayers and 3D ex vivo sebaceous glands that mimic acne-prone skin with the possibility of measuring lipid overproduction and metabolic activity and evaluating 3D bioimaging of virulent phylotype *C. acnes* inoculation. The specific localization of *C. acnes* to the active regions of the sebaceous glands and hair follicles is new and of great importance, as it allows accurate research. This model will allow us to develop actives in skin dysbiosis conditions, as well as investigate skin microbiota, which are of increasing importance in acne management and skin care strategies.

## 6. Patents

No patent resulting from the work is reported in this manuscript.

## Figures and Tables

**Table 1:** The growth conditions and stability kinetics of the strains during the study.

STRAIN	MEDIA	GROWTH	INCUBATIONS	CELL NUMBERS	COUNT POINT
*Staphylococcus epidermidis* CIP28.61	TSA agar	Aerobic	37 °C for 24/48 h * 5% CO_2_	1.0 × 10^7^	2 h/6 h/24 h/48 h
*Cutibacterium acnes* DSM 1897-Type IA	COS agar	Anaerobic	37 °C for 48/72 h * 5% CO_2_	1.0 × 10^7^	2 h/6 h/24 h/48 h

* For optimal enumeration, strains were grown in the time period appropriate for that type according to the experience of the microbiology laboratory, and the initial growth media agar was similarly chosen.

**Table 2:** Effects of bacterial concentration on ex vivo sebaceous gland cultures. Different concentrations were evaluated to determine their suitability for optimal culture conditions to the allow survival of glands and the evaluation of differences between conditions. TSA and COS agar conditions were standard microbiology laboratory procedures. No IC = no bacterial inocula were added to cultures. The 1 × 10^4^ evaluation test was used for *S. epidermidis*, and the 1 × 10^5^ standard test was used for *C. acnes* microbiological evaluations during the evaluation.

Condition	TSA (UFC/mL)	Average TSA	COS (UFC/mL)	Average COS
***S.epidermidis*** ** + *C.acnes* 1E5**	3.20 × 10^5^	**1.87 × 10^8^**	5.50 × 10^5^	**4.37 × 10^8^**
	1.90 × 10^8^		6.20 × 10^8^	
	3.0 × 10^8^		6.90 × 10^8^	
***S.epidermidis*** ** + rinse at 2 h**	1.99 × 10^8^	**1.46 × 10^8^**		
	2.03 × 10^8^			
	3.70 × 10^7^			
***S.epidermidis*** ** + rinse at 2 h + *C.acnes* 1E5 after rinse**	2.06 × 10^8^	**1.72 × 10^8^**	3.70 × 10^8^	**2.71 × 10^8^**
	2.59 × 10^8^		3.90 × 10^8^	
	5.10 × 10^7^		6.90 × 10^8^	
***S.epidermidis*** ** + rinse at 6 h**	5.90 × 10^8^	**4.57 × 10^8^**		
	5.40 × 10^8^			
	2.41 × 10^8^			
***S.epidermidis*** ** + rinse at 6 h + *C.acnes* 1E5 after rinse**	2.30 × 10^8^	**7.76 × 10^7^**	5.30 × 10^8^	**1.79 × 10^8^**
	1.10 × 10^6^		3.50 × 10^6^	
	1.78 × 10^6^		2.60 × 10^6^	
***S.epidermidis*** ** 1E4**	8.50 × 10^8^	**7.10 × 10^8^**		
	5.40 × 10^8^			
	7.40 × 10^8^			
***C.acnes*** ** 1E5**			1.30 × 10^9^	**1.09 × 10^9^**
			1.38 × 10^9^	
			5.80 × 10^8^	
**No IC**	>3.00 × 10^4^	**>3.00 × 10^4^**	>3.00 × 10^4^	**>3.00 × 10^4^**
	>3.00 × 10^4^		>3.00 × 10^4^	
	>3.00 × 10^4^		>3.00 × 10^4^	

## Data Availability

Supplementary Materials, further examples of the results, videos of the colonization of bacteria and certain protocols are available from the corresponding author.

## Supplementary Materials

The following supporting information can obtained from the authors or may be downloaded via this link: https://www.mdpi.com/article/10.3390/microorganisms11092183/s1, Video S1: Laser scanning confocal microscopy projection video of a sebaceous gland labeled with nuclei coloring (blue) and MC5R (green) and colonized with *S. epidermidis*; *C. acnes* (red); Video S2: Laser scanning confocal microscopy z-stack composite video of a sebaceous gland labeled with nuclei coloring (blue) and MC5R (green) colonized with *S. epidermidis*; *C. acnes* (red).
